# Microbiological Quality Assessment of Frozen Fish and Fish Processing Materials from Bangladesh

**DOI:** 10.1155/2016/8605689

**Published:** 2016-02-25

**Authors:** Sohana Al Sanjee, Md. Ekramul Karim

**Affiliations:** ^1^Department of Microbiology, Faculty of Biological Sciences, University of Chittagong, Chittagong 4331, Bangladesh; ^2^Environmental Biotechnology Division, National Institute of Biotechnology, Ganakbari, Ashulia, Dhaka 1349, Bangladesh

## Abstract

The present study aims at the microbiological analysis of export oriented frozen fishes, namely, Jew fish, Tongue Sole fish, Cuttle fish, Ribbon fish, Queen fish, and fish processing water and ice from a view of public health safety and international trade. Microbiological analysis includes the determination of total viable aerobic count by standard plate count method and enumeration of total coliforms and fecal coliforms by most probable number method. The presence of specific fish pathogens such as* Salmonella* spp. and* Vibrio cholerae* were also investigated. The TVAC of all the samples was estimated below 5 × 10^5^ cfu/g whereas the total coliforms and fecal coliforms count were found below 100 MPN/g and 10 MPN/g, respectively, which meet the acceptable limit specified by International Commission of Microbiological Specification for Food. The microbiological analysis of water and ice also complies with the specifications having TVAC < 20 cfu/mL, and total coliforms and fecal coliforms count were below the limit detection of the MPN method. Specific fish pathogens such as* Salmonella* sp. and* V. cholerae* were found absent in all the samples under the investigation. From this study, it can be concluded that the investigated frozen fishes were eligible for export purpose and also safe for human consumption.

## 1. Introduction

Fish and fishery products are not only nutritionally important but also important in global trade as foreign exchange earner for a number of countries in the world [1]. Fisheries and aquaculture sectors have become the second most important contributors in export earnings of Bangladesh, providing about 3.74% in national GDP, 2.7% in export earnings, and 22.23% in agriculture sector [2]. Due to wide range of global market including USA, UK, Japan, Belgium, Netherlands, Thailand, Germany, China, France, Canada, Spain, and Italy, the export of frozen fish, dry fish, and salted and dehydrated fish is increasing day by day from Bangladesh. Though there are 129 fish processing industries in Bangladesh, only 62 plants have EU approval. So it is very important to maintain the quality of the frozen fish for its acceptance in international trade as well as avoiding the health problems of consumers.

Fish are of great concern for export earnings because of their higher nutritive value such as high protein content, with little or no carbohydrate and fat value. But fish may be contaminated at various stages of transport, handling, and processing. This contamination may be related to the raw materials, personnel, and processing tools such as forklifts through leakage, insect, and pest harborage. Additionally, seafood can become contaminated during storage and processing [3, 4]. Contamination may be caused by foodborne pathogens which are naturally present in aquatic environments, such as* Vibrio* spp., or derived from sewage contaminated water such as* Salmonella* spp. [5]. Consumption of these contaminated fish may cause infection or intoxication to the consumers.

*Vibrio cholerae* is responsible for the third-highest number of shellfish-related illnesses, after noncholera* Vibrio* spp. and Norwalk viruses [6]. Toxigenic Ol (epidemic biotype) infections are associated with profuse, watery diarrhea whereas nontoxigenic, non-Ol biotype (except O139) infections result in septicaemia and mild gastroenteritis. In contrast to* Vibrio* spp., the incidence of* Salmonella* infections due to seafood consumption is still low compared with salmonellosis associated with other foods. However, detection of* Salmonella* spp. in seafood cannot be skipped as it is responsible for most of the foodborne diseases or gastroenteritis characterized by diarrhea, abdominal cramp, vomiting, nausea, and fever. According to Centers for Disease Control and Prevention,* Salmonella* is the leading cause of bacterial foodborne illness causing approximately 1.4 million nontyphoidal illnesses, 15,000 hospitalizations, and 400 deaths in the USA annually [7].

Water and ice quality is also an important factor for good quality fish, because water and ice used for fish processing may contaminate the whole processing plant. EU advised Bangladesh Government to implement the Hazard Analysis Critical Control Point (HACCP) in the processing of frozen fishes [8]. So it is important to find out the quality of fish we consume as well as of the frozen fish which are exported.

Therefore, the present study was carried out to investigate the microbiological quality of the marine frozen fishes for raising food safety concern and promoting international trade. This study also investigated the microbiological quality of water and ice, as these factors were intimately correlated with the fish processing and preservation.

## 2. Materials and Methods

### 2.1. Study Area

The study was carried out in “Taj Munir Fish Preserver Ltd.,” situated at the port city Chittagong of Bangladesh. The study was conducted from June 2011 to February 2014. During the study period, total viable aerobic count, total coliforms and fecal coliforms counts, and presence of pathogenic organisms (namely,* Salmonella* spp. and* Vibrio cholerae*) of public health significance from the frozen fishes (storage temperature −20°C) such as Jew fish (*Argyrosomus hololepidotus*), Tongue Sole fish (*Cynoglossus broadhursti*), Cuttle fish (*Sepia officinalis*), Ribbon fish (*Lepturacanthus savala*), Queen fish (*Scomberoides commersonnianus*), and water and ice which were used during the processing of samples were investigated. All the frozen fishes were gutted and organoleptically good enough to carry out further bacteriological analysis. Sampling was done each year at three months' interval, namely, June, October, and February. During study periods, triplicate samples for each fish species as well as for ice and water samples were analyzed independently.

### 2.2. Chemicals and Media

Pure and analytical grade chemicals purchased from BDH Chemicals Ltd., England; Merck, Germany; and Siga Chemical Co. Ltd., USA, were used throughout the study including media preparation. All the media and media ingredients such as beef extract and peptone that are used throughout the study were from Scharlau, Spain.

For the enumeration of coliforms and fecal coliforms, Lauryl Tryptose Broth (LTB) and 2% Brilliant Green Bile Broth (BGLBB) were used, respectively. Bismuth Sulfite Agar (BSA) and Xylose Lysine Deoxycholate (XLD) agar were used for the detection of* Salmonella* spp. whereas Thiosulfate Citrate Bile Salt (TCBS) agar and Cellobiose, Polymyxin, and Colistin (CPC) were used for the detection of* V. cholerae.*

### 2.3. Fish Samples Preparation

All glassware was sterilized (121°C, 15 psi, 20 minutes) before use. Triplicate fish samples (each about 25 g) of each fish type were measured separately in an analytical balance (Model: ML204/01, Mettler Toledo, Switzerland) in aseptic condition and then dissolved into about 225 mL buffered peptone water (BPW) and blended for (30–60) seconds in a sterilized blender machine [8]. Each fish sample was blended and homogenized separately.

### 2.4. Water and Ice Collection

The water (namely, WS_1_, WS_2_, and WS_3_) and ice (namely, IS_1_, IS_2_, and IS_3_) were collected in 1-liter sterilized container from different location. The collected samples were preserved in the refrigerator (4°C), when analysis was delayed for more than 3 hours.

### 2.5. Enumeration of TVAC of Fish, Water, and Ice

Total viable aerobic bacteria of fish, water, and ice were enumerated by standard plate count (SPC) method [9]. For the enumeration of TVAC, serial dilution of each sample was carried out up to 10^−5^ dilutions with 9 mL sterilized 0.1% peptone water, from which an aliquot of 1 mL of each dilution was aseptically poured into duplicate sterile Petri plate, and sterile melted (around 40–45°C) Plate Count Agar was poured over it, rotated clockwise-anticlockwise, allowed to solidify, and finally incubated at inverted position at 37°C for 24–48 hours.

After incubation, the plates having well spaced colonies (30–300) were used for counting and the colonies were counted by a colony counter (Stuart Scientific, UK). Total viable aerobic count per mL or per g was calculated by multiplying the average number of colonies per plate by reciprocal of the dilution and expressed as colony forming units (cfu) per mL or per g of sample [10].

### 2.6. Enumeration of Total Coliforms of Fish

Most Probable Number (MPN) method is used for the quantitative estimation for coliform [11]. Serial dilution of the samples was prepared as described earlier. Nine test tubes containing about 9 mL Lauryl Tryptose Broth (LTB) with inverted Durham's tube were sterilized. Three test tubes were inoculated with 1 mL from 10^−1^ dilution, another three test tubes were inoculated from 10^−2^ dilution, and the remaining three test tubes were inoculated from 10^−3^ dilution. The inoculated tubes were incubated at 37° for 48 hours. Test tubes showing positive results (gas production in Durham's tube) were counted and recorded as presumptive positive for coliforms.

### 2.7. Enumeration of Fecal Coliforms (Presumptive* E. coli* Test) of Fish

About one loopful from each gas positive LTB was inoculated into test tube of sterilized BGLBB and a test tube of sterilized 10 mL Tryptone Broth and then incubated at 44.5° ± 0.5°C for 48 hours. After incubation, gas production was recorded and 2-3 drops of Kovac's reagent were added to each of the positive tubes. A positive indole reaction in Tryptone Broth that has produced cherry red color indicates the presence of* E. coli.* The positive gas production tubes were recorded and results were compared using Most Probable Number (MPN) chart to determine the total fecal coliforms number (*E. coli*) per gram [12].

### 2.8. Enumeration of Total Coliforms and Fecal Coliforms in Water and Ice

About 50 mL of water was inoculated to 50 mL of sterilized LTB (double strength) in one mega test tube whereas about 10 mL of water was inoculated in five test tubes containing 5 mL of sterile LTB (double strength) and about 1 mL of water was also inoculated in five test tubes containing 5 mL of sterile LTB (single strength) in each of the test tubes. All of the test tubes contained inverted Durham's tubes. After incubation at 37°C for 24–48 hours, the positive results were recorded which indicate total coliforms count (MPN/100 mL). About one loopful from the gas positive tubes was inoculated in BGLBB with inverted Durham's tubes and sterilized Tryptone Broth and then incubated at 44.5° ± 0.5°C for 24–48 hours. After incubation, 2-3 drops of Kovac's reagent were added to the Tryptone Broth and cherry red color indicated total fecal coliforms/100 mL. The total coliforms and fecal coliforms of ice samples were also enumerated similarly.

### 2.9. Detection of* Salmonella* spp

*Salmonella* spp. were detected following the procedure as described by Andrews and Hammack [13]. About 25 g samples were dissolved in about 225 mL of sterilized buffered peptone water (BPW), blended, and incubated at 37°C for 16–20 hours. About 10 mL from the incubated BPW culture was selectively enriched into the 100 mL sterilized Selenite Cystine Broth and incubated again at 37°C for 24–48 hours. After incubation, 1 loopful inoculum from the selective enrichment culture was streaked onto the preincubated BSA and XLD agar plate. Typical* Salmonella* spp. produce pink colonies with or without black centers on XLD agar and brown, grey, or black colonies on BSA agar. Then the suspected colonies were identified by their cultural, morphological, and biochemical characteristics, namely, TSI (Triple Sugar Iron), Urease test, MR-VP test, Oxidase test, Citrate test, fermentation of carbohydrates (Glucose, Sucrose, Arabinose, Mannose, Mannitol, and Inositol), and Decarboxylase (Lysine, Arginine, and Ornithine) following the taxonomic guides of* Bergey's Manual of Determinative Bacteriology*, 8th ed. [14]. All cultures giving biochemical reactions were confirmed by agglutination test with* Salmonella* polyvalent (O) somatic antisera [15].

### 2.10. Detection of* Vibrio cholera*

*Vibrio cholera* was detected following the procedure as described by Kaysner and Angelo [16]. About 25 g samples were blended with 225 mL sterilized Alkaline Peptone Water (APW) and incubated at 37°C for 16–18 hours. Then 1 loopful inoculum from the APW culture was streaked on the preincubated TCBS and CPC agar plate and incubated at 37° for 24 hours. Typical colonies of* V. cholerae* on TCBS agar are large, yellow, and smooth whereas on CPC agar they are small, smooth, opaque, and green to purple in color. Then the suspected colonies were identified by their cultural, morphological, and biochemical characteristics, namely, Oxidase test, fermentation of carbohydrates (Glucose, Sucrose, Arabinose, Mannose, Mannitol, and Inositol), and Decarboxylase test (Lysine, Arginine, and Ornithine) according to the taxonomic guides of* Bergey's Manual of Determinative Bacteriology*, 8th ed. [14]. Finally the* V. cholerae* were confirmed by agglutination test using polyvalent* V. cholerae* (O) antiserum [15].

## 3. Results and Discussion

Fish and seafoods hold an important position as a food component for a large section of world population [17]. In Bangladesh, the export of fish and fishery products has gained a remarkable position in the earnings of foreign currencies in the last few years. So, maintenance of appropriate quality of the products is regarded as vital for achieving desired success in the global trade of this product.

Jew fish (*A. hololepidotus*), Queen fish (*S. commersonnianus*), Tongue Sole fish (*C. broadhursti*), Ribbon fish (*L. savala*), and Cuttle fish (*S. officinalis*) are the most commonly exported marine fishes from Bangladesh. The maximum microbiological limit for the TVAC which separates the good quality products from bad quality is 5 × 10^5^ cfu/g [18]. The TVAC of the studied samples ranged from 2.8 × 10^5^ to 4.9 × 10^5^ cfu/g which was below the maximum acceptable limit. So all the samples of each type of the fish meet the acceptable limit specified by ICMSF which points out the good quality of the frozen fishes.

**Figure 1:**
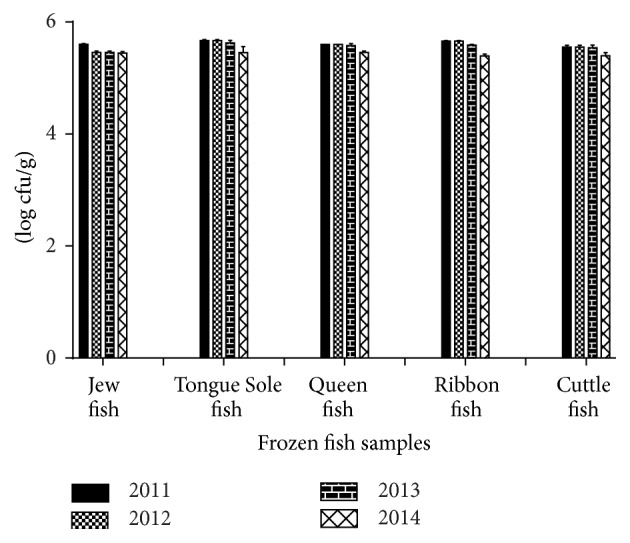
Total viable aerobic count (log cfu/g) of the frozen fish samples. The data were representatives of the three independent experiments using triplicate samples and mean ± SD values were expressed.

Figure 1 showed the TVAC of all frozen fish samples from which it was observed that the density of total aerobic bacteria detected in the Tongue Sole fish samples was comparatively higher than all of the fishes whereas the lowest bacterial count was observed in the samples of Jew fish. Loads of bacteria in fish samples decreased gradually over time in all of the fishes. This may be due to the aseptic processing and handling, proper sampling, trained personnel, improved storage conditions, and increased awareness for preservation.

**Figure 2:**
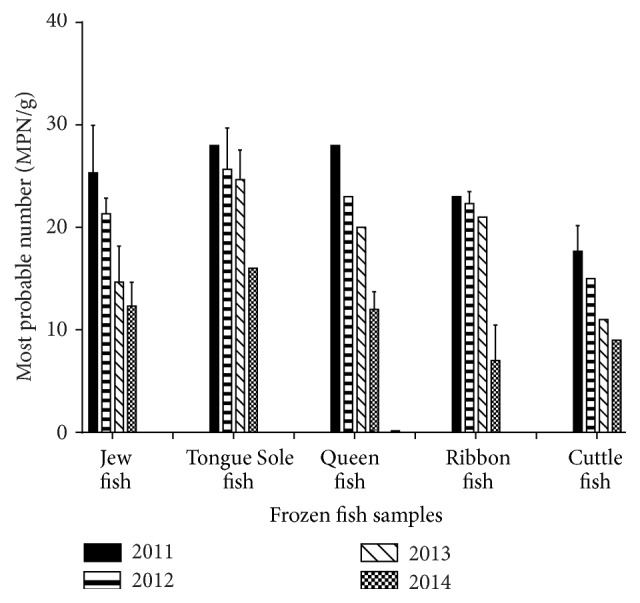
Total coliforms count (MPN/g) of frozen fish samples. The data were representatives of the three independent experiments using triplicate samples and mean ± SD values were expressed.

**Figure 3:**
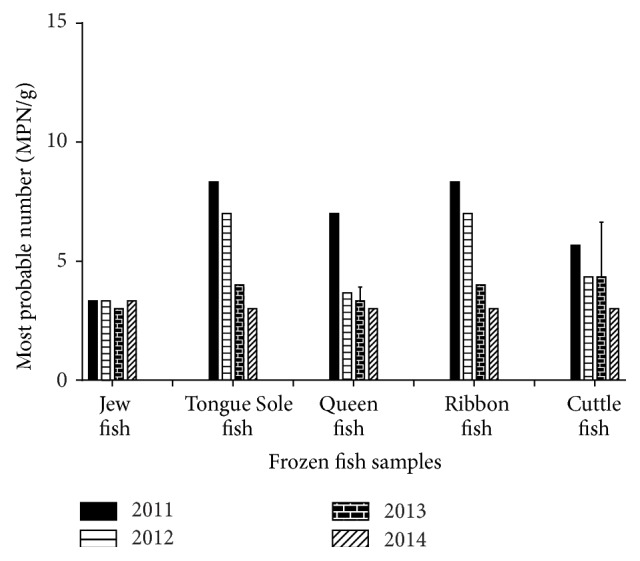
Total fecal coliforms count (MPN/g) of frozen fish samples. The data were representatives of the three independent experiments using triplicate samples and mean ± SD values were expressed.

The acceptable limits of total coliforms (TC) and fecal coliforms (FC) for fresh and frozen fish are <100 MPN/g and <10 MPN/g, respectively [18]. The presence of TC is indicator of sewage contamination which may also occur during different processing steps such as transport and handling. Moreover, the contamination may also be caused by the water used for washing or icing [19]. The more accurate indicator of fecal contamination is fecal coliforms that is* E. coli* [20]. The lower number of coliforms can be beneficial for pointing out the effectiveness of safety procedures during processing and handling [21]. In the present study, the total coliforms count ranged from 5 MPN/g to 28 MPN/g and fecal coliforms count was from 3 MPN/g to 8.3 MPN/g. Figures 2 and 3 showed the highest number of coliforms and fecal coliforms bacteria in Tongue Sole fish whereas the lowest count was observed in the samples of Jew fish, respectively. Our study revealed that all the samples were within the recommended limits which indicated that the samples were collected from pollution-free water and also the food processors and handlers maintained aseptic conditions throughout the processing.

**Figure 4:**
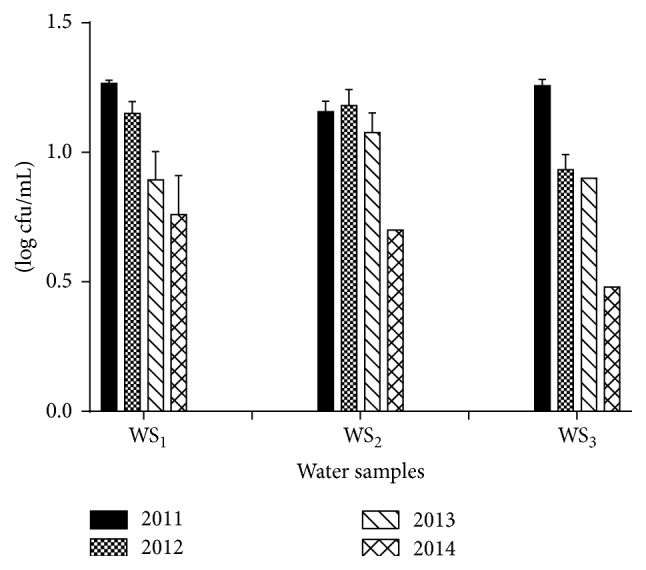
Total viable aerobic count (log cfu/mL) of water samples. The data were representatives of the three independent experiments using triplicate samples and mean ± SD values were expressed.

**Figure 5:**
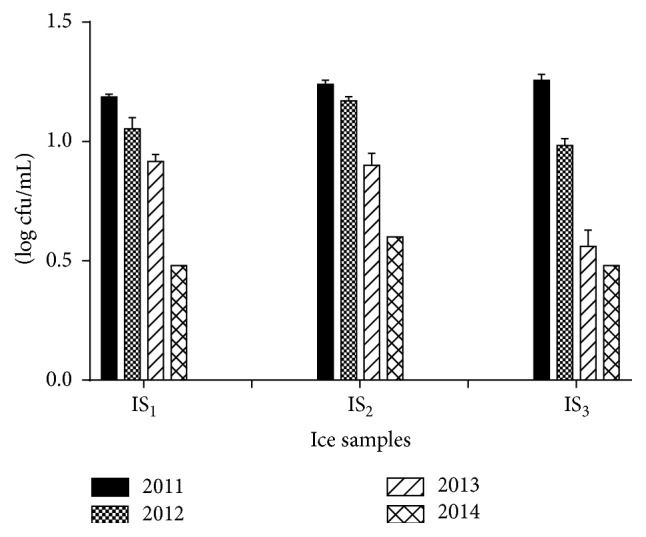
Total viable aerobic count (log cfu/mL) of ice samples. The data were representatives of the three independent experiments using triplicate samples and mean ± SD values were expressed.

Water and ice are the most important factor for the processing of exported fish. These two factors contribute to determining and maintaining of the standard quality of the frozen fishes. Figures 4 and 5 showed the TVAC of water and ice samples, respectively, over the study period. It was found that TVAC of water samples and ice samples ranged from 3 to 18 cfu/mL. Significant reduction of TVAC for both water and ice samples was observed over the time period. The total coliforms and fecal coliforms count were found absent for both samples. Hence, our study revealed that all the tested samples complied with the recommended limit specified by ICMSF, that is, TVAC having <20 cfu/mL, and coliforms and fecal coliforms count were below the limit detection of the MPN method. This may be due to the advanced and improved facilities for water and ice purification, treatment, and handling.

Seafood infections are caused by variety of bacteria, viruses, and parasites. According to Centers for Disease Prevention and Control (CDC), during 1973 to 2006, 188 outbreaks of seafood-associated infections, causing 4,020 illnesses, 161 hospitalizations, and 11 deaths, were reported to the Foodborne Disease Outbreak Surveillance System. Most of these seafood-associated outbreaks (143 (76.1%)) were due to a bacterial agent; 40 (21.3%) outbreaks had a viral etiology; and 5 (2.6%) had a parasitic cause. According to the report,* Vibrio* were the most commonly reported cause of seafood-associated outbreaks where toxigenic* V. cholerae* caused 3 outbreaks and 10 illnesses without deaths and non-O1, non-O139* V. cholerae* caused 4 outbreaks and 12 illnesses without deaths, whereas* Salmonella* was responsible for 18 outbreaks, 374 illnesses, and 28 hospitalizations during the study period [22].

Recently CDC reported that about 62 people were infected with* Salmonella* Paratyphi B variant L (+) tartrate (+) (formerly known as* Salmonella* Java) from 11 states of USA related to the consumption of frozen raw tuna. The infection was characterized by diarrhea, fever, and abdominal cramps after 12–72 hours' exposure without paratyphoid fever, enteric fever, or typhoid fever [23].

Although in Bangladesh, foodborne illness related to fresh or frozen seafood consumption has not been traced yet or data on this issue is still lacking. In this context, microbiological analysis of frozen fish and fishery products seems to be an important issue. This type of study generates scientific information which would help in preventing and controlling future outbreaks related to seafood consumption. According to the rule of International Association of Microbiology Society, fresh and frozen fish should possess neither* Vibrio* spp. nor* Salmonella* spp. The investigated frozen samples were of good quality as all the samples were free from these pathogenic microorganisms.

Bacterial growth in the frozen fishes is one of the main causes of food spoilage or contamination of fish. Hence, the microbiological analysis of the frozen fish samples and fish processing materials (water and ice) acts as the indicator of fish quality determination. The present study reported that all of the fish samples along with the materials meet the standard levels suggested by ICMSF which indicated that aseptic and proper hygienic conditions were maintained properly throughout all steps such as catching, landing, transportation, processing, handling, and preservation.

## 4. Conclusion

Although seafood is part of a healthful diet, its consumption is not out of risk. Worldwide continued outbreaks of seafood-associated infections have rendered the existing control strategies questionable. An understanding of the etiologic agents, seafood commodities associated with illness, and mechanisms of contamination that are amenable to control is thus necessary for the prevention of seafood-associated infection outbreaks. Coordinated efforts from government sector and private industry together with federal agencies are urgently needed in this context. There is a need for routine surveillance systems using pathogen-specific techniques to avoid any future outbreaks. However, the current study revealed that microbiological quality of the investigated frozen fishes and fish processing materials (ice and water) was within the specified limit of ICMSF. So it can be concluded that these fishes were processed with properly treated pathogen-free water and ice and, finally, maintained at good storage condition. Hence, the investigated frozen fishes were qualified enough for export as well as human consumption from bacteriological point of view. The presence of viruses, parasites, viable but nonculturable (VBNC) state of the pathogenic bacteria, and biochemical parameters such as histamine risk might be a problem in frozen fish products which is the limitations of this study. Beyond ICMSF, in order to comply with more stringent indigenous quality standards of the exporting countries, these quality parameters must be taken into consideration.
